# Overcoming the H4K20me3 epigenetic barrier improves somatic cell nuclear transfer reprogramming efficiency in mice

**DOI:** 10.1111/cpr.13519

**Published:** 2023-06-15

**Authors:** Zhihui Liu, Weiguo Wang, Yuhan Xia, Yuan Gao, Zhisong Wang, Mingyang Li, Giorgio Antonio Presicce, Liyou An, Fuliang Du

**Affiliations:** ^1^ Jiangsu Key Laboratory for Molecular and Medical Biotechnology, College of Life Sciences Nanjing Normal University Nanjing China; ^2^ ARSIAL Rome Italy; ^3^ Xinjiang Key Laboratory of Biological Resources and Genetic Engineering, College of Life Science and Technology Xinjiang University Urumqi China

## Abstract

Epigenetic reprogramming during fertilization and somatic cell nuclear transfer (NT) is required for cell plasticity and competent development. Here, we characterize the epigenetic modification pattern of H4K20me3, a repressive histone signature in heterochromatin, during fertilization and NT reprogramming. Importantly, the dynamic H4K20me3 signature identified during preimplantation development in fertilized embryos differed from NT and parthenogenetic activation (PA) embryos. In fertilized embryos, only maternal pronuclei carried the canonical H4K20me3 peripheral nucleolar ring‐like signature. H4K20me3 disappeared at the 2‐cell stage and reappeared in fertilized embryos at the 8‐cell stage and in NT and PA embryos at the 4‐cell stage. H4K20me3 intensity in 4‐cell, 8‐cell, and morula stages of fertilized embryos was significantly lower than in NT and PA embryos, suggesting aberrant regulation of H4K20me3 in PA and NT embryos. Indeed, RNA expression of the H4K20 methyltransferase *Suv4‐20h2* in 4‐cell fertilized embryos was significantly lower than NT embryos. Knockdown of *Suv4‐20h2* in NT embryos rescued the H4K20me3 pattern similar to fertilized embryos. Compared to control NT embryos, knockdown of *Suv4‐20h2* in NT embryos improved blastocyst development ratios (11.1% vs. 30.5%) and full‐term cloning efficiencies (0.8% vs. 5.9%). Upregulation of reprogramming factors, including *Kdm4b*, *Kdm4d*, *Kdm6a*, and *Kdm6b*, as well as ZGA‐related factors, including *Dux*, *Zscan4*, and *Hmgpi*, was observed with *Suv4‐20h2* knockdown in NT embryos. Collectively, these are the first findings to demonstrate that H4K20me3 is an epigenetic barrier of NT reprogramming and begin to unravel the epigenetic mechanisms of H4K20 trimethylation in cell plasticity during natural reproduction and NT reprogramming in mice.

## Graphical abstract

H4K20me3 is an epigenetic barrier to NT reprogramming and *Suv4‐20h2* knockdown in NT embryos decreased H4K20me3 to levels comparable to those of fertilized embryos, overcoming the 2‐cell development block and improving the development ratio of NT embryos (blastocyst rate) and the efficiency of newborn cloned mice (cloned rate) by facilitating transcriptional reprogramming.

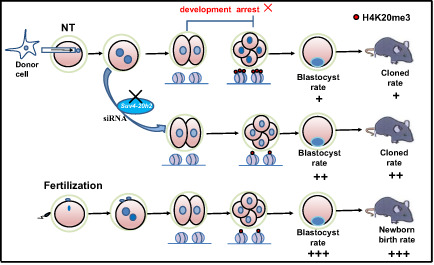

## 1 INTRODUCTION

Trimethylation of lysine 20 of histone H4 (H4K20me3) is an important posttranslational epigenetic modification that is generated when the histone methyltransferase, suppressor of variegation 4–20 homologue 2 (Suv4‐20h2， also known as KMT5C),^1^, ^2^ adds a methyl group to H4K20me2.^3^ H4K20me3 is a hallmark of silenced heterochromatic regions and is a key player in the epigenetic regulation of genomic integrity.^4^ H4K20me3 is enriched in constitutive heterochromatin, centromeres, telomeres, and regions of silenced genes.^2^, ^3^, ^4^ Silencing pathways induce H3K9me3 and H4K20me3 at constitutive heterochromatin, and the interaction of Suv4‐20h2 and heterochromatin protein 1 isoforms can establish H3K9me3 and H4K20me3 at pericentric heterochromatin.^3^ TGF‐β signalling alters H4K20me3 status via miR‐29 to induce cellular senescence.^5^ H4K20me3 modification is dynamically regulated by Suv4‐20h2 and histone demethylase homologue of Rad23 proteins (hHR23, called Rad23 in mice), which regulate the appearance of heterochromatin late in development and subsequent transcription of repetitive elements.^6^, ^7^ Conversely, H4K20me3 also co‐localizes with activating histone modifications at transcriptionally dynamic regions in embryonic stem cells.^8^ Wongtawan et al. reported that H4K20me3 is a late heterochromatin marker that is specifically located at the perinuclear rings and is undetectable during preimplantation development in mice.^7^ These studies indicate that early preimplantation mouse embryos lack constitutive heterochromatin, which is thought to result in an immature chromatin state that supports the cell plasticity necessary for development.^7^, ^9^

Somatic cell nuclear transfer (NT) is a biological platform to study the mutual nucleus–cytoplasm interaction.^10^ Mice are often used as a mammalian model to study the molecular and epigenetic reprogramming of NT. The first cloned mouse was generated by Wakayama et al.^11^ with only 2%–2.8% of transferred NT embryos developing to term. Low full‐term embryo development after NT in mice is often attributed to aberrant epigenetic regulation of gene expression at reprogramming resistant regions of the genome due to epigenetic barriers, such as H3K9me3,^12^ H3K27me3,^13^, ^14^ and H3K4me3,^15^ epigenetic modifications, DNA methylation,^16^, ^17^ and aberrant *Xist* gene activation.^18^ It is unknown whether H4K20me3 contributes to the epigenetic barriers of NT. During NT reprogramming, a highly differentiated somatic nucleus is transferred into a recipient oocyte at metaphase II (MII) for nuclear remodelling, including nuclear envelope breakdown and subsequent premature chromosome condensation (PCC), which may be induced by a maturation‐promoting factor in the oocyte cytoplasm.^19^, ^20^ The epigenetic modification pattern of H4K20me3 during NT reprogramming is unknown in mice. Therefore, it is important to explore H4K20me3‐modified heterochromatin after NT to elucidate the role of H4K20me3 in NT reprogramming.

In this study, we extensively investigated the dynamic pattern of H4K20me3 modification during preimplantation developmental stages of fertilized, parthenogenetic activation (PA), and NT embryos. We evaluated the pattern, localization, and intensity of H4K20me3 as well as the expression of factors that regulate H4K20me3 modification, including the histone methyltransferase *Suv4‐20h2* and the demethylase *Rad23b*. Finally, we knocked down *Suv4‐20h2* in NT embryos to study the function of H4K20m3 as an epigenetic barrier to preimplantation embryo development in mice. Altogether, our study reveals that aberrant modification of H4K20me3 in NT embryos is an alternative molecular regulator and epigenetic barrier to nuclear reprogramming.

## 2 MATERIALS AND METHODS

### 2.1 Reagents

Chemicals, unless otherwise noted, were purchased from Sigma‐Aldrich.

### 2.2 Ethical approval

All animal care and use procedures were approved by the Animal Care and Use Committee of Nanjing Normal University (IACUC‐20201209) and performed according to guidelines of the US National Institutes of Health.

### 2.3 Superovulation and collection of mouse oocytes and fertilized embryos

To collect embryos at different pronucleus (PN) stages (PN1‐PN5), superovulation was induced in sex‐matured ICR female mice (6–8‐weeks‐old, Yangzhou University) by injection of 7.5 IU pregnant mare serum gonadotropin (PMSG, San‐Sheng Pharmaceutical Co. Ltd., Ningbo, China), followed by 10 IU human chorionic gonadotropin (hCG, Ningbo Second Hormone Factory) 47 h later. Subsequently, mice were mated overnight, and copulation plugs were checked the following morning. Female donors were sacrificed at 14–20 h post‐hCG injection, and zygotes were collected from the oviduct. Similarly, embryos at 2‐cell, 4‐cell, 8‐cell, morula, and blastocyst stages were collected on 1.0, 2.0, 2.5, 3.0, and 4‐days post coitum, respectively. For MII oocyte collection, superovulated females were not mated with the males, and instead, oocytes were collected 14 h after hCG injection. Germinal vesicle (GV) oocytes were collected from the follicles of ovaries of untreated sex‐matured female mice. Oocytes and embryos were cultured in vitro (IVC) in 50 μL droplets of K‐modified simplex optimized medium (KSOM, Millipore) supplemented with 4% bovine serum albumin (BSA) at 37°C and 5% CO_2_ in a humidified atmosphere prior to immunofluorescent (IF) microscopy with H4K20me3 antibody.

### 2.4 Donor cell preparation, NT, PA, and embryo IVC

Fresh cumulus cells were collected from B6D2F1 mice purchased from Yangzhou University, China, and used as donor cells for NT, as described by Wakayama et al.^11^ Briefly, cumulus cells were removed from cumulus‐oocyte‐complexes (COC) using 0.1% hyaluronidase and washed several times in HEPES‐ Chatot‐Zimomek‐Bavister medium (HCZB). Then, the cumulus cells were resuspended with HCZB containing 3% PVP (polyvinyl pyrrolidone, 360 kDa) and stored at 4°C prior to NT.

NT micromanipulations were performed as described by Liu et al.^21^ Briefly, B6D2F1 oocytes were collected from oviducts 14 h after hCG injection. Oocytes were enucleated with a blunt Piezo‐driven pipette on an Olympus inverted microscope (Olympus IX71, Japan) in HCZB supplemented with 5 μg/mL cytochalasin B (C6762). Donor cell nuclei were transferred into enucleated oocytes, and then activated by 5 h incubation in 10 mM SrCl_2_ in Ca^2+^‐free CZB and 5 μg/mL cytochalasin B. Pronuclear formation was evaluated, and NT oocytes were thoroughly washed and subsequently cultured in KSOM at 37°C under 90% N_2_, 5% O_2_, and 5% CO_2_ for 72 h. NT 2‐cell, 4‐cell, and 8‐cell embryos as well as morulae and blastocysts were collected at 24, 48, 60, 72, and 96 h post‐IVC, respectively.

IVC of PA oocytes was performed in the same manner used for NT embryos. PA 2‐cell, 4‐cell, and 8‐cell embryos as well as morulae and blastocysts were collected at 20, 40, 60, 72, and 96 h post‐IVC, respectively.

### 2.5 Immunofluorescence (IF) microscopy

Oocytes (GV, MII), donor cells, fertilized embryos, PA embryos, and NT embryos at different stages (1‐cell, 2‐cell, 4‐cell, 8‐cell, morula, and blastocyst) were fixed with fresh 4% paraformaldehyde in DPBS for 15 min, washed and stored in DPBS at 4°C until immunostaining. After washing three times in DPBS, permeabilization was performed by treatment with 0.5% Triton‐X 100 for 15 min and washing in 0.2% DPBS/Tween 20 (PBST) for 30 min at room temperature. Blocking of non‐specific binding was performed by incubation in 2% BSA DPBS for 1 h at room temperature. Immunostaining was performed by incubation of primary monoclonal antibody against H4K20me3 (ABclonal, Wuhan, China, Cat. A2372, dilution 1:100) and Rad23b (ABclonal, Cat. A21586, dilution 1:200) overnight at 4°C. After washing for 15 min in PBST, samples were incubated with secondary antibody Alexa Fluor 488 goat anti‐rabbit IgG (ABclonal, Cat. AS053, dilution 1:300) for 1 h. Oocytes, fertilized, PA, and NT embryo samples were washed in PBST for 30 min and subsequently stained with 4,6‐diamidino‐2‐phenylindole (DAPI; 100 ng/mL; D9564) for 15 min and mounted on slides. Images of H4K20me3 staining were collected using fluorescence microscopy (Olympus BX53, Japan) with an exposure time of 500 ms. ImageJ (v1.8.0; National Institutes of Health) was used to perform H4K20me3 intensity analysis of chromosomes at the MII phase and of nuclei at the 1‐cell, 2‐cell, 4‐cell, 8‐cell, morula, blastocyst stages as follows. The corrected cumulative optical density of the nuclear region of an embryo was calculated as the cumulative optical density of the total nuclear region minus the background optical density (the nuclear area multiplied by the average optical density per unit area of the oocyte/embryo). Briefly, images were first converted to 16‐bit greyscale, and the background value was eliminated using the background subtraction function in Image J. Nuclear intensities of integrated fluorescent images were measured and compared among PA, NT, and fertilized embryos.

### 2.6 Quantitative RT‐PCR

For quantitative RT‐PCR experiments, varying numbers of oocytes and embryos were pooled as follows: 150 oocytes at either GV or MII stages, 150 embryos at the 1‐cell stage (fertilized, PA), 80 embryos at the 2‐cell stage (fertilized, PA), 50 embryos at the 4‐cell stage (fertilized, PA), 30 embryos at the 8‐cell stage (fertilized, PA), 30 morulae (fertilized, PA), and 20 blastocysts (fertilized, PA). Separate pools of cells were lysed by incubation in 50 μL lysis buffer (Abm, Richmond, British Columbia, Canada) at 37°C for 10 min, and 5 μL stopping buffer (Abm) was added to terminate the reaction. cDNA was synthesized using 5X All‐in‐One RT MasterMix (Abm, Cat: G916), according to the manufacturer's instructions. β‐actin was used as an endogenous control for normalization. Primers are listed in Table S1. Quantitative RT‐PCR was performed using EvaGreen 2 × qPCR MasterMix‐ROX (Abm). Relative mRNA transcript levels were calculated using the $2^{-∆∆\mathrm{Ct}}$ method.^22^ Gene expression levels of the GV group were normalized to 1.0 for comparison of expression between different stages of fertilized and PA embryos. Due to the limited number of NT embryos, RNA expression by RT‐PCR was not evaluated at different developmental stages.

### 2.7 Microinjection of small interfering RNA

Short interfering RNA (siRNA) sequences against *Suv4‐20h2* that were previously reported^23^ were purchased from Sangon Biotech, The siRNA pair used was as follows: forward strand 5‐GGCUAGUGGUCAGUCAUGGUUCUAU‐3 and reverse strand 5‐AUAGAACCAUGACUGACCACUAGCC‐3. A non‐specific siRNA pair was used as a control (forward strand 5‐GGAUGGAUGAAAUGGUAUGGAGAAA‐3 and reverse strand: 5‐UUUCUCCAUACCAUUUCAUCCAUCC‐3). siRNA micromanipulations were performed after 5 h activation (Act) of the reconstructed embryos using 1 μg/μL siRNA. Approximately 10 pL of the siRNA mixture was injected into the cytoplasm of each reconstructed embryo in HCZB medium. The injected embryos were then cultured in KSOM medium, and 4‐cell NT embryos were collected after 36 h to detect RNA levels of *Suv4‐20h2* by RT‐PCR.

### 2.8 Embryo transfer

Embryos injected with siRNA and control siRNA were cultured in KSOM medium for 18 h. Subsequently, 12–15 embryos at the 2‐cell stage were transferred into each side of the oviducts of pseudopregnant ICR females. Caesarean sections were performed on pregnant mice 19 days post embryo transfer prior to birth. The reason that siRNA and control siRNA embryos were transferred into either side of the oviducts of same recipient was to avoid the term development variation of embryos in different recipients during pregnancy.

### 2.9 Statistical analysis

Percentages of NT, PA, and fertilized embryos were analysed using SPSS 18.0 (IBM, Chicago, IL, USA). Percentages of each replicate were arcsine‐transformed and subjected to one‐way ANOVA. Means were compared using Fisher's least significant difference test. Fluorescence intensity and RNA expression were analysed using SPSS version 18.0, and one‐way ANOVA was used to evaluate the differences in H4K20me3 intensity among NT, PA, and fertilized embryos at different stages of development. Statistical significance was defined as *p* < 0.05.

## 3 RESULTS

### 3.1 H4K20me3 dynamics in 1‐cell zygotes, PA oocytes, and NT embryos

**FIGURE 1:**
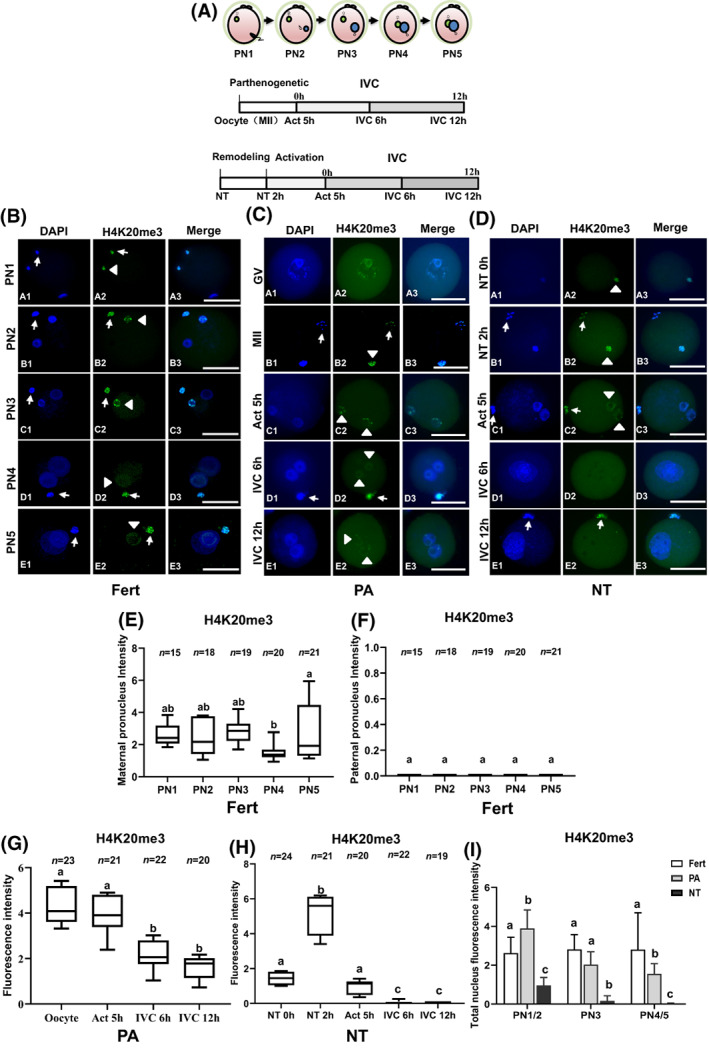
H4K20me3 dynamics in 1‐cell in vivo‐fertilized, PA, and NT embryos. (A) Upper panel: Fertilized mouse zygotes were classified as PN1, PN2, PN3, PN4, or PN5 according to the relative position between the maternal (M) and paternal (P) pronuclei (PN) in the zygote. Middle panel: Timing of PA and in vitro culture (IVC) of oocytes. Bottom panel: Timing of nuclear transfer (NT), PA, and IVC of cloned embryos. (B) H4K20me3 staining in maternal PN (PN1–PN5, shown as arrowhead) in five categorized groups displayed ring‐like H4K20me3 staining but was not observed in paternal PN. The polar bodies of zygotes showed H4K20me3 signal at metaphase plates (shown as arrows). (C). GV oocytes carried dot‐shaped heterochromatin staining (A1–A3). After completion of meiosis, MII oocytes (B1–B3) showed H4K20me3 signal at metaphase plates of both oocytes (arrowhead) and polar bodies (arrow). After activation for 5 h (Act 5 h), oocytes showed two PPN with spotty H4K20me3 staining (C1–C3), and subsequently ring‐like H4K20me3 staining after 6 h (IVC 6 h, D1–D3) and 12 h IVC (IVC 12 h, E1–E3). Arrows indicate H4K20me3 signal in the polar bodies. (D) NT oocytes showed weak H4K20me3 staining of donor nuclei just after completing NT (NT 0 h, A1–A3). After remodelling for 2 h (NT 2 h), the injected nuclei underwent PCC, and strong H4K20me3 staining was localized on PCC (B1–B3). After activation for 5 h (act 5 h), NT oocytes showed two PPN carrying ring‐like H4K20me3 signals (arrowheads, C1–C3). However, after IVC for 6–12 h, H4K20me3 was undetectable in PPN (IVC 6 h, D1–D3; IVC 12 h, E1–E3). Arrows indicate the metaphase plate in polar bodies. (E)–(F) H4K20me3 staining was only present in maternal PN (2.4–4.5) and was completely absent in paternal PN (0) of zygotes at stages PN1–PN5. H4K20me3 intensity was 2.4 at PN4, which was lower than that at PN5 (4.5) (*p* < 0.05). (G) MII oocytes carried the highest H4K20me3 intensity (5.2). After activation, H4K20me3 intensity dramatically dropped to 4.1 activation (Act 5 h) and continued to decline to 2.1 (IVC 6 h) and 1.6 (IVC 12 h), respectively (*P* < 0.05). (H) Immediately post‐nuclear injection (NT 0 h), donor cell nuclear H4K20me3 intensity was 1.4 (NT 0 h), which increased to 5.7 after oocyte nuclear remodelling for 2 h (NT 2 h), indicating oocyte cytoplasm possessed H4K20 methylation function. However, after activation, NT oocytes showed significantly reduced H4K20me3 intensities of 1.0 (Act 5 h), 0.07 (IVC 6 h), and 0.09 (IVC 12 h). (I) The H4K20me3 signal of NT embryos was significantly lower than that of fertilized and PA embryos at similar PN stages. Different letters indicate statistically significant differences between groups (*p* < 0.05). Scale bars = 50 μm.

In this experiment with three replicates, the number of zygote, PA, and NT embryos was allocated as 93, 101, and 106, respectively (Figure 1). Mouse zygotes were classified from PN1 to PN5 according to the position of the maternal and paternal pronuclei^24^ (Figure 1A). IF microscopy showed that H4K20me3 was present in maternal pronuclei and polar bodies (PB) but absent in paternal pronuclei from PN1 to PN5 (Figure 1B). GV oocytes showed nuclear H4K20me3 staining, and MII oocytes had apparent H4K20me3 staining in both the PB and metaphase plate (Figure 1C). Immediately after PA (5 h), PA oocytes showed two pronuclei carrying H4K20me3, which was dispersed in both pronuclei as spotty punctiform (Figure 1C, Act 5 h). Following 6 and 12 h IVC, PA oocytes displayed H4K20me3 staining localized as peri nucleolar rings when two pseudo‐pronuclei (PPN) moved together (Figure 1C, IVC 6 h, and IVC 12 h). In NT oocytes, donor nuclei underwent PCC (Figure 1D) after 2 h remodelling. H4K20me3 staining was observed in donor cell nuclei (NT 0 h), PCC chromosomes (NT 2 h), and two PPN (Act 5 h) after nuclear remodelling (2 h) and activation (5 h); however, H4K20me3 staining was absent in PPN of NT embryos post 6 h (IVC 6 h) and 12 h IVC (IVC 12 h).

Intensity analyses revealed that zygotes possessed a dominant maternal pronuclear H4K20me3 staining pattern from PN1 to PN5 whereas paternal nuclei did not show detectable H4K20me3 staining in any pronuclear stages. In addition, a similar intensity of H4K20me3 staining was observed in maternal pronuclei from PN1 to PN5, with the exception of PN4, which demonstrated significantly lower H4K20me3 staining intensity than that of PN5 (*p* < 0.05, Figure 1E). In contrast, the highest H4K20me3 signal intensity was recorded in MII oocytes but was reduced in the PPN of activated PA oocytes post‐activation (Act 5 h) and was further decreased after 6–12 h IVC (IVC 6 h, IVC 12 h), respectively (*p* < 0.05). NT oocytes were remodelled in KSOM medium for 2 h and subjected to activation for 5 h. After activation, NT oocytes underwent IVC for 12 h and were then collected for immunostaining. Interestingly, H4K20me3 intensity in donor nuclei significantly increased in PCC after remodelling for 2 h (NT 2 h) (Figure 1H) but decreased in PPN after activation (Act 5 h) and was undetectable after 6 and 12 h IVC (Figure 1H). We further defined and compared H4K20me3 intensity of similar 1‐cell stages of fertilized, PA, and NT embryos, according to the size and location of pronuclei. We found that NT embryos at PN1/2, PN3, and PN4/5 stages exhibited the lowest H4K20me3 intensities compared to similar stages in fertilized and PA embryos (Figure 1I).

### 3.2 Dynamic H4K20me3 modification pattern in fertilized, PA, and NT mouse embryos

**FIGURE 2:**
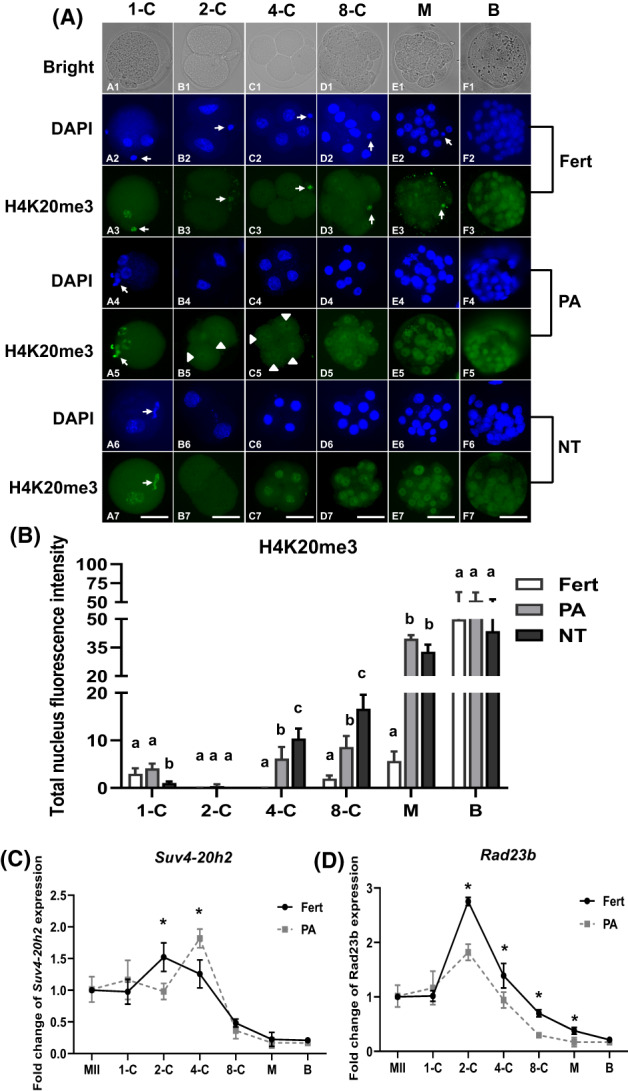
H4K20me3 dynamics of in vivo‐fertilized, PA, and NT embryos and *Suv4‐20h2* and *Rad23b* RNA expression in fertilized and PA embryos at different developmental stages. (A) Fertilized embryo panels: Zygotes showed H4K20me3 in maternal PN (arrowhead) and the polar bodies (arrow) but not in paternal PN (A2–A3). However, H4K20me3 staining was absent at the 2‐cell (*n* = 28, B2–B3) and 4‐cell (*n* = 25, C2–C3) stages and reappeared at the 8‐cell (D2–D3), morula (E2–E3), and blastocyst (F2–F3) stages. PA embryo panels: After activation, oocytes carried H4K20me3 staining inside two PPN (A4–A5), and H4K20me3 staining intensity decreased in 2‐cell embryos (arrowheads, B4–B5). H4K20me3 staining intensity gradually increased throughout the 4‐cell (C4–C5), 8‐cell (D4–D5), morula (E4–E5), and blastocyst (F4–F5) stages. NT embryo panels: After NT remodelling and 5 h activation, donor nuclei were remodelled into two oocyte PPNs and carried H4K20me3 staining (A6–A7), but H4K20me3 staining was absent in the 2‐cell stage (B6–B7). H4K20me3 staining gradually increased at the 4‐cell (C6–C7), 8‐cell (D6–D7), morula (E6–E7), and blastocyst (F6–F7) stages (arrow indicating metaphase H4K20me3 staining in polar bodies of embryos). Scale bar = 60 μm. (B) Values of a and b within cell stages indicate statistically significant differences (*p* < 0.05). The H4K20me3 mean intensity of fertilized (*n* = 20) and PA (*n* = 22) 1‐cell embryos was significantly higher than NT (*n* = 21) embryos. In contrast, the intensities of fertilized 4‐cell (*n* = 25), 8‐cell (*n* = 25), and morula (*n* = 26) embryos were significantly lower than those of PA (4‐cell, *n* = 24; 8‐cell, *n* = 22) and NT (4‐cell, *n* = 20; 8‐cell, *n* = 16) embryos. (C) Left: The level of *Suv4‐20h2* RNA began to increase at the 2‐cell stage and decreased at the 4‐cell stage of fertilized embryos, whereas the level of *Suv4‐20h2* RNA increased in PA embryos at the 4‐cell stage and decreased at the 8‐cell stage. *Suv4‐20h2* expression levels of fertilized 4‐cell embryos were significantly lower than those of PA embryos but were significantly higher at the 2‐cell stage compared with PA embryos. Right: Levels of *Rad23b* RNA began to increase at the 1‐cell stage of both fertilized and PA embryos and reached maximal levels at the 2‐cell stage. *Rad23b* expression gradually dropped at the 4‐cell, 8‐cell, morula, and blastocyst stages. *Rad23b* expression in fertilized 2‐cell, 4‐cell, and 8‐cell embryos and morulae was significantly higher than those of PA embryos. * Indicates a significant difference between fertilized and PA embryos of the same cell stages (*p < 0.05*). 1‐C, 2‐C, 4‐C, 8‐C, M, and B represent 1‐cell, 2‐cell, 4‐cell, 8‐cell, morula, and blastocyst stages, respectively.

In this experiment with four replicates, the number of fertilized, PA and NT embryos was allocated as 146, 139, and 122, respectively (Figure 2A, B). Perinuclear rings were stained with H4K20me3 in maternal PN but not in paternal PN of 1‐cell zygotes. Interestingly, H4K20me3 signals were undetectable in 2‐cell and 4‐cell nuclei, and H4K20me3 staining reappeared in 8‐cell embryos and was distributed in most morula and blastocyst cells (Figure 2A). In the PA group, a spotty pattern of H4K20me3 signal was first observed in PPN PA oocytes after activation (Figure 2A) and was followed by perinuclear ring staining in PPN of cultured PA oocytes (IVC 6 h and IVC 12 h) (Figure 2A). Additionally, weak H4K20me3 staining was observed in 2‐cell and 4‐cell PA embryos, and H4K20me3 signal increased in the 8‐cell, morula, and blastocyst stages (Figure 2A). Cumulus donor cell nuclei initially exhibited H4K20me3 staining (Figure 1D) that was maintained in injected donor nuclei remodelled into larger PPN at the 1‐cell stage of NT (Figure 2A). Surprisingly, H4K20me3 staining disappeared from the nuclei of 2‐cell NT embryos, but it was apparent at perinuclear rings in 4‐cell, 8‐cell, morula, and blastocyst NT stages (Figure 2A).

We then compared H4K20me3 staining intensity between fertilized, PA, and NT embryos at different developmental stages (Figure 2B). We observed significantly lower H4K20me3 intensity in NT embryos compared to fertilized and PA embryos at the 1‐cell stage (*p* < 0.05), whereas no significant differences were observed at the 2‐cell stage (Figure 2B). Interestingly, NT and PA embryos demonstrated higher intensities of H4K20me3 compared with fertilized embryos at 4‐cell, 8‐cell, and morula stages (*p* < 0.05). However, there was no significant difference in H4K20me3 intensity at the blastocyst stage between the three types of embryos (Figure 2B). Overall, NT and PA embryos shared a significantly higher H4K20me2 pattern than that in fertilized embryos at 4‐cell, 8–cell and morula stages, respectively (Figure 2B).

### 3.3 Expression profiles of *Suv4‐20h2* and Rad23b in mouse fertilized PA, and NT embryos

In this experiment with three replicates, the number of fertilized, and PA embryos was allocated as 1080, and 1530, respectively (Figure 2C, D). To investigate potential causes of the H4K20me3 elevation observed in NT and PA embryos, we analysed the RNA expression levels of the histone methyltransferase *Suv4‐20h2* and the demethylase *Rad23b* at different preimplantation stages. Due to the limited scale of cloned (NT) embryo production, only fertilized and PA embryos were included in quantitative RT‐PCR experiments. We found that *Suv4‐20h2* RNA expression levels were similar in oocytes and at the 1‐cell zygotes post‐fertilization but clearly increased at the 2‐cell stage, followed by gradual, but significant, decreases at the 4‐cell, 8‐cell, morula, and blastocyst stages, indicating a peak in *Suv4‐20h2* expression or zygotic genome activation (ZGA) at the 2‐cell stage in fertilized embryos. In PA embryos, *Suv4‐20h2* RNA peak ZGA expression occurred at the 4‐cell stage (Figure 2C). Interestingly, *Suv4‐20h2* levels were significantly higher in PA embryos than in fertilized embryos at the 4‐cell stage but were significantly lower at the 2‐cell stage in PA embryos compared with fertilized embryos. PA and fertilized embryos showed similar *Suv4‐20h2* expression levels at 1‐cell, 8‐cell, morula, and blastocyst stages (Figure 2C).

Both fertilized and PA embryos demonstrated *Rad23b* ZGA at the 2‐cell stage (Figure 2D). *Rad23b* expression levels were similar between 1‐cell fertilized and PA embryos. Interestingly, we observed a significant increase in *Rad23b* RNA expression in 2‐cell, 4‐cell, 8‐cell, and morula fertilized embryos compared to PA embryos, with an approximately two‐fold increase in fertilized embryos over PA embryos at the 2‐cell stage (Figure 2D *p* < 0.05). In contrast, *Rad23b* expression was similar in fertilized and PA blastocysts. Likewise, Rad23b protein expression was similar between PA and NT embryos. However, a significant increase of Rad23b expression was observed in fertilized embryos compared to that in PA and NT embryos (Figure S1, *p* < 0.05).

Overall, these results indicate that *Suv4‐20h2* was more highly expressed in 4‐cell PA embryos, whereas *Rad23b* was consistently higher from the 2‐cell to morula stages of fertilized embryos (Figure 2C, D). This dynamic imbalance of *Suv4‐20h2* (higher methyltransferase) and *Rad23b* (lower demethylase) that occurred during PA reprogramming may contribute to the elevation of H4K20me3 in both NT and PA embryos compared with fertilized embryos.

### 3.4 Knockdown of *Suv4‐20h2* improves NT embryonic development

**FIGURE 3:**
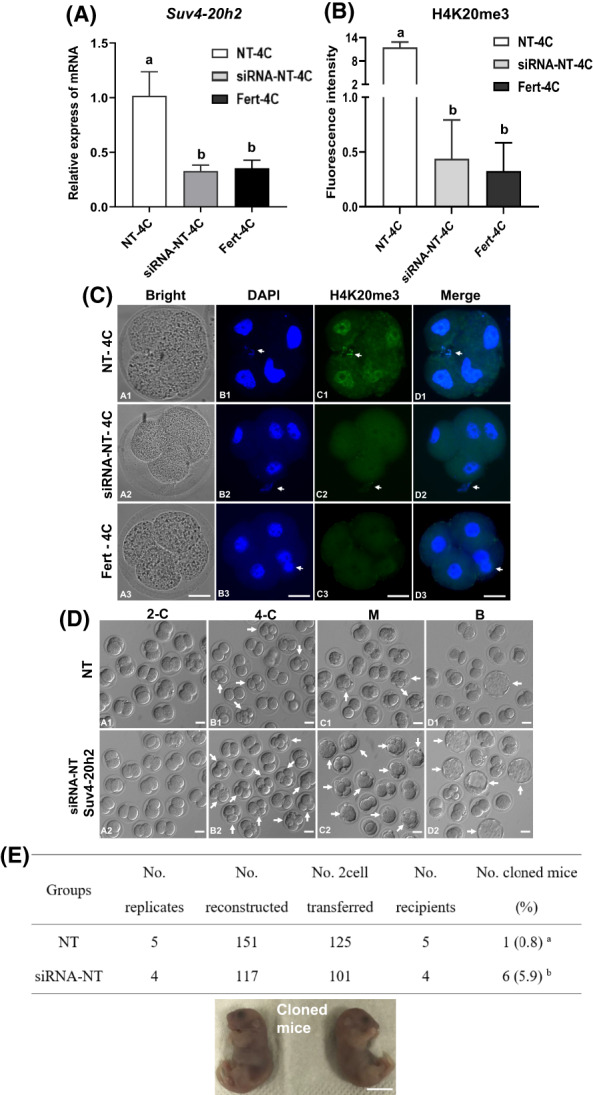
Knockdown of *Suv4‐20h2* influences the development ability of NT embryos and cloning efficiency in mice. (A) Values of a and b within two kinds of embryos indicate statistically significant differences (*p* < 0.05). The level of *Suv4‐20h2* RNA was reduced significantly in NT 4‐cell embryos after treatment with *Suv4‐20h2* siRNA and returned to the level of fertilized 4‐cell embryos. (B) Values of a and b within cell stages indicate statistically significant differences (*p* < 0.05). Fluorescence intensity analysis showed that knockdown of *Suv4‐20h2* reduced the level of H4K20me3 modification significantly in NT 4‐cell embryos and returned H4K20me3 levels to those of fertilized embryos. Different letters in the same column indicate significant differences (*p* < 0.05). (C) Fluorescence staining showed that *Suv4‐20h2* siRNA injection in reconstructed 1‐cell NT embryos reduced the H4K20me3 modification in NT 4‐cell embryos (*n* = 18) significantly. siRNA‐NT embryos (*n* = 22) exhibited similar patterns of H4K20me3 as fertilized 4‐cell embryos (*n* = 26). Scale bar = 20 μm. (D) Knockdown of *Suv4‐20h2* in NT embryos improved the development ratio of 4‐cell (B1–B2), morula (C1–C2), and blastocyst (D1–D2) stages significantly. Scale bar = 50 μm. (E) Representative *Suv4‐20h2* siRNA‐treated cloned newborn mice 19 days after embryo transfer. Scale bar = 1 cm.

In this experiment, the total number of fertilized, and NT embryos was allocated as 178, and 499, respectively (Figure 3A–D). Owing to the high expression of *Suv4‐20h2* in NT embryos, we examined the effect of *Suv4‐20h2* knockdown on preimplantation development by microinjecting NT embryos with *Suv4‐20h2* siRNA or its control RNA after activation. After 36 h, 4‐cell embryos were collected for quantitative RT‐PCR and IF staining to detect *Suv4‐20h2* expression and H4K20me3 levels, respectively. We found that *Suv4‐20h2* RNA levels in siRNA‐NT embryos were reduced to the level of fertilized embryos and were significantly lower than control NT embryos at the 4‐cell stage (Figure 3A). Moreover, we observed that H4K20me3 staining intensities in siRNA‐NT embryos were weaker than those of control NT embryos, and its pattern was similar in siRNA‐NT and fertilized embryos at the 4‐cell stage (Figure 3C, C1–C3). Next, we compared total nuclear fluorescence intensities for H4K20me3 at the 4‐cell stage between fertilized, siRNA‐NT and control NT embryos. We found that H4K20me3 levels in siRNA‐NT embryos were lower than those of control NT embryos at the 4‐cell stage (Figure 3B *p* < 0.05). Additionally, siRNA‐NT embryos showed similar H4K20me3 intensities when compared with fertilized embryos at the 4‐cell stage (Figure 3B *p* > 0.05). To evaluate developmental potential, we cultured embryos for 24, 48, 72, or 96 h and quantified the number of embryos that developed into 2‐cell, 4‐cell, morula, or blastocyst stages, respectively. We observed an approximately three‐fold increase in siRNA‐NT embryos at the 4‐cell, morula, and blastocysts stages compared to control NT embryos (Figure 3D). When the cleavage rate was compared between the siRNA‐NT and control NT groups, there were no differences at the 2‐cell stage (83.7% vs. 82.4%); however, the developmental rate was significantly higher in siRNA‐NT embryos at the 4‐cell (54.0% vs. 25.2%), morula (50.4% vs. 22.1%), and blastocyst (30.5% vs. 11.1%) stages compared to control NT embryos (Table 1, *p* < 0.05).

**TABLE 1:** Knockdown of *Suv4‐20h2* improves the development ability of NT embryos.

Groups	Replicates	Embryos	2‐cell (%)	4‐cell (%)	Morula (%)	Blastocyst (%)
NT	3	79	65 (82.4 ± 6.5)^a^	16 (25.2 ± 6.3)^a^	14 (22.1 ± 8.2)^a^	7 (11.1 ± 4.1)^a^
siRNA‐NT	3	89	75 (83.7 ± 6.0)^a^	40 (54.0 ± 10.1)^b^	37 (50.4 ± 9.3)^b^	23 (30.5 ± 4.6)^b^

*Note*: a, b values with different superscripts within columns indicate statistically significant differences (*p* < 0.05). After injection of *Suv4‐20h2* siRNA and control siRNA, embryos were cultured in KSOM medium for 20, 36, 72, and 96 h to detect ratios of 2‐cell, 4‐cell, morula, and blastocyst stages, respectively. NT: somatic cell nuclear transfer. siRNA‐NT: somatic cell nuclear transfer and treatment with siRNA targeting *Suv4‐20h2*.

### 3.5 Knockdown of *Suv4‐20h2* improves the cloning efficiency of mice

The total number of si‐RNA‐NT and control NT embryos was allocated as 117 and 151, respectively, (Figure 3E, F). To further investigate the effect of *Suv4‐20h2* knockdown on post‐implantation development, we transferred NT embryos generated from cumulus cell donors of B6D2F1 mice at the 2‐cell stage into the oviducts of pseudopregnant female ICR mice. Caesarian sections were performed on day 19 to determine cloning success rates. Importantly, 5.9% (6/101) of transferred *Suv4‐20h2* siRNA‐injected 2‐cell NT embryos developed to term, which was significantly higher than that of the control NT group (0.8%, 1/125, *p* < 0.05) (Figure 3E). Together, these results demonstrate that H4K20me3 in donor somatic cells is an epigenetic barrier for oocyte‐mediated genomic reprogramming, and removal of the H4K20me3 barrier by *Suv4‐20h2* siRNA injection at very early stages of NT improves the efficiency of mouse reproductive cloning.

### 3.6 Knockdown of *Suv4‐20h2* accelerates the transcription of reprogramming and ZGA genes

**FIGURE 4:**
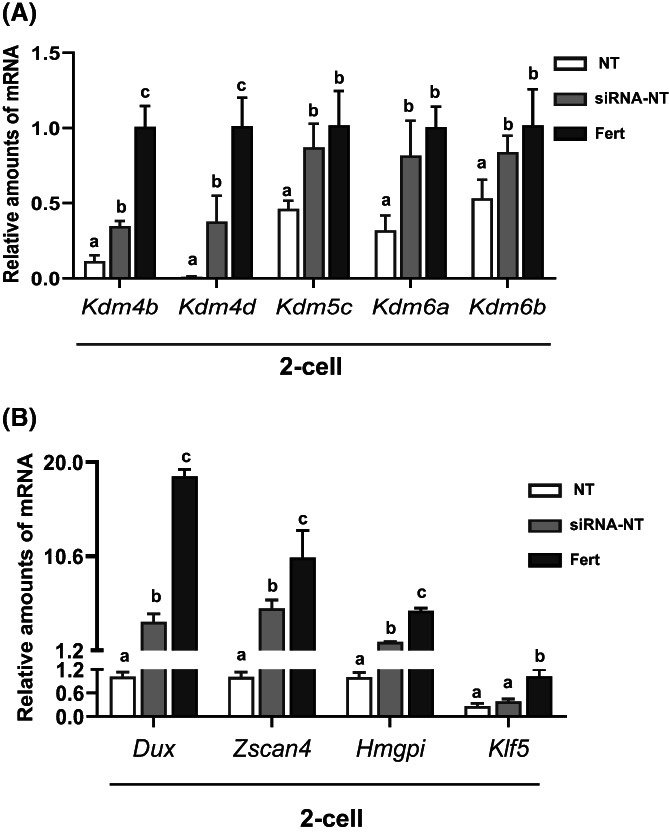
Differential gene expression in fertilized, NT, and siRNA‐NT 2‐cell embryos. (A) Values of a, b, and c within two types of embryos indicate statistically significant differences (*p* < 0.05). Expression of two genes involved in reprogramming, *Kdm4b*, and *Kdm4d*, was upregulated in NT 2‐cell embryos with *Suv4‐20h2* siRNA treatment compared with control NT 2‐cell embryos. Expression levels of *Kdm4b* and *Kdm4d* in NT and siRNA‐NT 2‐cell embryos were significantly lower compared to fertilized 2‐cell embryos (*p* < 0.05). The RNA levels of *Kdm5c*, *Kdm6a*, and *Kdm6b* were upregulated significantly in fertilized embryos compared with NT embryos at the 2‐cell stage (*p* < 0.05). (B) Expression of ZGA‐related genes (*Dux*, Zscan*4*, *Hmgpi*) was upregulated in NT 2‐cell embryos with *Suv4‐20h2* siRNA treatment, whereas expression levels in NT and siRNA‐NT 2‐cell embryos were all lower than in fertilized 2‐cell embryos (*p* < 0.05). Values of a, b, and c within cell stages indicate statistically significant differences (*p* < 0.05).

Previous studies have identified several reprogramming genes, including *Kdm4b*, *Kdm4d*, *Kdm5c*, *Kdm6a*, and *Kdm6b*, that are important for NT reprogramming and play important roles in somatic cell reprogramming processes.^12^, ^15^, ^25^, ^26^ Therefore, we evaluated the RNA expression levels of these reprogramming genes in three groups of 2‐cell embryos (siRNA‐NT, control NT, and fertilized). We found that *Suv4‐20h2* knockdown significantly upregulated the RNA levels of *Kdm4b* and *Kdm4d* compared with the control NT group, though both levels were still lower than those of the fertilized group (Figure 4A *p* < 0.05). More importantly, not only were the RNA levels of *Kdm5c*, *Kdm6a*, and *Kdm6b* significantly upregulated in the siRNA‐NT group compared to the control NT group, but the RNA levels were also restored to the levels of fertilized embryos at the 2‐cell stage (Figure 4A *p* > 0.05).

To investigate the underlying molecular mechanism of the proposed H4K20me3 epigenetic barrier, we evaluated the effect of *Suv4‐20h2* knockdown on mouse ZGA in three types of 2‐cell embryos (siRNA‐NT, control NT, and fertilized). We selected several genes that are activated at specific ZGA stages in fertilized mouse embryos, including *Dux*， *Zscan4*， *Hmgpi*, and *Klf5*.^27^, ^28^, ^29^ Quantitative RT‐PCR analysis revealed that *Suv4‐20h2* knockdown in NT 2‐cell embryos significantly upregulated the levels of genes involved in ZGA, including *Dux*, *Zscan4*, and *Hmgpi*, compared with the control NT group (*p* < 0.05) (Figure 4B). *Klf5*, another gene involved in ZGA, levels were not different between the siRNA‐NT and control NT groups. Importantly, the RNA levels of ZGA‐related genes were still lower in siRNA‐NT embryos than in fertilized embryos at the 2‐cell stage (Figure 4B).

## 4 DISCUSSION

In this study, we demonstrate dynamic profiles of H4K20me3 in IVC fertilized preimplantation mouse embryos (zygotes, 2‐cell, 4‐cell, 8‐cell, morula, and blastocysts) and in PA and NT embryos during nuclear reprogramming. The presence of H4K20me3 in oocytes at meiotic GV and MII suggests that it plays a role in remodelling oocyte nuclear chromatin structure and in regulating gene expression during meiosis. After fertilization, localization of H4K20me3 was pericentric or in ring‐like nucleolar structures in PB and maternal, but not paternal pronuclei, which is consistent with previous reports.^7^, ^9^ However, we found that H4K20me3 was dynamic across preimplantation development from 2‐cell to blastocyst stages, which is distinctly different from previous findings.^7^, ^9^ Wongtawan et al. reported that H4K20me3 was undetectable from 2‐cell to blastocyst stages and was limited to peri‐implantation embryos and embryonic stem cells,^7^ which is supported by an additional report of H4K20me3 absence in 2‐cell embryos.^9^ Notably, these previous reports did not include the H4K20me3 signature in oocytes (GV and MII). We suggest that the discrepancy in H4K20me3 patterns is due to the stringency of antibodies used in the different studies. More specifically, the primary antibody for H4K20me3 used in previous study was from Millipore (USA),^7^ whereas the primary antibody for H4K20me3 used in this study was from ABclonal (Canada, distributed in China). In our study, the H4K20me3 signature was primarily observed as a dotty distribution near pericentromeric heterochromatin regions in GV nuclei (Figure 1C), accumulated at the metaphase plate in MII oocytes (Figure 1C), and developed into periphery ring‐like nucleolar regions in maternal PN (Figure 1B). In addition, the nuclear structures of PB in MII oocytes and fertilized embryos (from zygote to morula and blastocyst) all exhibited strong H4K20me3 staining, which was absent in paternal PN of zygotes from PN1 to PN5. These observations further confirm the specificity of the antibody used in these studies. Additionally, quantitative RT‐PCR revealed that methyltransferase *Suv4‐20h2* mRNA was highly expressed at the 2‐cell stage (Figure 2C), which is indicative of ZGA in fertilized mouse embryos. In contrast, Eid et al. reported reduced *Suv4‐20h2* RNA expression in 2‐cell embryos compared with zygotes.^9^ They hypothesized that the lack of conventional constitutive heterochromatin in zygotes and 2‐cell embryos was linked to chromatin dynamics that support a higher level of developmental plasticity.^9^ Additionally, H4K20me3 is thought to be a late heterochromatin marker in embryonic development.^7^ However, our results contradict previous findings and suggest instead that H4K20me3 is dynamically maintained across all stages of preimplantation. Our results demonstrate that low levels of H4K20me3 remain in the heterochromatin of early embryos and may contribute to the remodelling and maintenance of the embryonic nuclear heterochromatin architecture. As an evolutionarily conserved constitutive heterochromatin mark, H4K20me3 is a specific feature of centromeres and telomeres and is a repressive regulator of gene expression that should be maintained in the heterochromatin of early embryos, as observed in GV and MII oocytes. However, we acknowledge that early preimplantation embryos possess a high degree of cell plasticity for cell lineage determination and cell commitment during embryonic development.

As a constitutive marker of heterochromatin, H4K20me3 is thought to reflect repressive conformations of chromatin structure.^3^, ^7^, ^15^ Repressive chromatin is interspersed with active chromatin in the mammalian genome.^30^, ^31^ Thus, the pattern of H4K20me3 is evolutionarily conserved and regulates position‐effect variegation.^3^ H4K20me3 patterns and dynamics are thought to be controlled by the functional interplay and/or balance between histone methyltransferases, such as Suv4‐20h2,^7^ and histone demethylases, such as Rad23b.^6^ Our quantitative RT‐PCR studies indicated that *Suv4‐20h2* and *Rad23b* RNA expression levels were stable in both MII oocytes and fertilized 1‐cell embryos, suggesting a balance of H4K20 trimethylation exists during meiosis and after fertilization. After completing meiosis, the chromosome set is condensed and remodelled into a high‐order structure as bivalent homologous chromosomes form synapses in prophase (i.e., GV phase) to MII. Indeed, H4K20me3 staining was observed in regions with strong DAPI staining, indicative of highly folded DNA at the metaphase plate in the MII phase (Figure 1C). As previously noted, a maternal pattern of H4K20me3 modification has been reported during meiosis.^7^ As the pronuclear stage is when paternal nuclei undergo dramatic replacement of protamine by histone proteins, it is reasonable to hypothesize that H4K20 trimethylation may be delayed in paternal nuclei. For example, additional time is likely to be necessary for posttranslational modification of new histone H4 proteins by histone methyltransferases, including SET8 and Suv4‐20h1, to generate H4K20me1 and H4K20me2 in paternal PN, which then requires additional methylation of H4K20me2 by Suv4‐20h2.^2^ Notably, H4K20me3 staining intensity was the lowest in the 2‐cell stage and increased as embryos developed to morula and blastocyst stages. *Suv4‐20h2* RNA levels were lowest at the 1‐cell zygote stage, implying that *Suv4‐20h2* RNA is rapidly consumed after fertilization. We observed ZGA of *Suv4‐20h2* at the 2‐cell stage, which may contribute to the maintenance of H4K20me3 in embryos during preimplantation development. *Rad23b* RNA levels were highest at the 2‐cell stage, indicating its ZGA at the 2‐cell stage as well (Figure 2D). We hypothesize that the balance between Suv4‐20h2 and Rad23b plays an important role in the dynamics of the H4K20me3 signature in cleaved embryos (2‐cell–8‐cell) as well as morulae and blastocysts.

The efficiency of NT is typically low, and our understanding of the underlying causes of low NT efficiency and the related inherent reprogramming mechanisms remains limited. Known barriers to cloning and reprogramming include incomplete global epigenetic modifications, such as H3K9me3,^12^ H3K27me3,^26^, ^32^ H3K4me3,^15^ imprinting genes,^33^ X‐chromosome inactivation,^18^ and DNA methylation.^17^ Importantly, some of these barriers can be overcome. For example, H3K9me3 reduction via either knockdown of *Suv39h1/2* or overexpression of the corresponding demethylase improves NT efficiency in both mice (Kdm4d)^12^ and humans (Kdm4a).^34^ The combined use of Kdm4b and Kdm5b (demethylase of H3K4me3) greatly improves cloned blastocyst and birth rates in mice.^15^ Furthermore, knockdown of DNA methyltransferases rescues re‐methylation defects in mouse NT embryos.^17^ Matoba et al. reported that reduced H3K9me3 histone modification by *Kdm4d* overexpression in NT embryos increased the cloning mice efficiency to 8.7%,^12^ while by both knockout of *Xist* and overexpression of *Kdm4d*, it was increased up to 23.5%.^32^ Gao et al. increased its efficiency to 11.1% by overexpression of *Kdm4b* and *Kdm5b* ^15^; and further by knockdown of *Dnmt3a/3b* to reduce DNA methylation in NT embryos, they increased cloning efficiency to 17.2%.^17^ Wang et al. demonstrated that overcoming the H3K27me3 imprinting barrier improved the cloning efficiency to 14.2%.^33^ In our study, the cloning efficiency was improved to 5.9% by overcoming the H4K20me3 histone modification barrier, indicating that H4K20me3 is a repressive modification to NT reprogramming and that overcoming multiple epigenetic modification barriers may be an effective strategy to improve total cloning efficiency. However, the role of H4K20me3 in oocyte reprogramming is largely not understood. We first investigated the H4K20me3 profile in PA mouse oocytes because NT embryos are activated in the same manner when an oocyte receives a differentiated somatic nucleus. We found that activated oocytes and PA embryos maintained H4K20me3 at nearly every stage from MII to blastocyst. The 2‐cell stage was the exception and demonstrated almost undetectable H4K20me3 staining, which gradually increased again as development progressed (Figure 2A). Donor nuclei demonstrated a weaker H4K20me3 signal than MII oocytes (Figure 1D). After transfer into oocyte cytoplasm, donor nuclei underwent a series of remodelling changes, including nuclear swelling, PCC (NT 2 h) during nuclear remodelling, and further PPN post‐activation (Act 5 h) (Figure 1D), reflecting the dramatic exchange or replacement of oocyte RNA and protein with those transcribed and translated from donor nuclear chromatin. H4K20me3 was absent in late 1‐cell (Figure 1D) and 2‐cell (Figure 2A) NT embryos, suggesting that *Suv4‐20h2* is rapidly depleted from the oocyte cytoplasm, especially post‐PA activation. H4K20me3 levels increased in NT embryos only after the 4‐cell stage, suggesting recovery of *Suv4‐20h2* expression in NT embryos. We also observed that the preimplantation development of NT embryos was inferior to that of PA embryos,^35^ indicating that NT embryos possess lower development potential. Furthermore, both PA and NT groups showed significantly lower development rates compared to fertilized embryos.^35^ We propose that this developmental disadvantage may be due to reprogramming barriers that exist in PA and NT embryos. H4K20me3 intensity in both PA and NT embryos was significantly higher than in fertilized embryos at 4‐cell, 8‐cell, and morula stages (Figure 2B), indicating higher methylation and lower demethylation of nuclear H4K20me3 in both PA and NT embryos. To correct this delay, knockdown of *Suv4‐20h2* or overexpression of *Rad23b* at the RNA or protein level in 1‐cell NT embryos could be performed to reduce H4K20me3. Indeed, we demonstrated that injection of *Suv4‐20h2* siRNA into NT embryos at the 1‐cell stage reduced H4K20me3 in NT embryos (Figure 3). Although *Suv4‐20h2* knockdown or *Rad23b* overexpression may achieve similar effects in NT embryo development, future studies are necessary to evaluate the competent developmental potential of NT embryos and the resulting cloning efficiency after genetic manipulation.

The developmental block often observed in 2‐cell IVC mouse embryos coincides with the timing of ZGA in embryo development, which is crucial for preimplantation embryo development and for an embryo to develop to term.^21^, ^27^, ^36^ *Suv4‐20h2* knockdown in NT embryos significantly upregulated the transcription levels of the zygotic genes *Dux*, *Zscan4*, and *Hmgpi* compared with control NT embryos but did not restore expression of these genes to the levels observed in fertilized 2‐cell embryos (Figure 4B). Nevertheless, upregulation of zygotic gene expression after *Suv4‐20h2* knockdown improves the development potential of NT embryos and cloning efficiency in mice. The reprogramming factors *Kdm4b*, *Kdm4d*, *Kdm5c*, *Kdm6a*, and *Kdm6b* demethylases are vital for NT remodelling and play important roles in NT processes.^12^, ^15^, ^25^ We found that *Suv4‐20h2* knockdown significantly upregulated the expression levels of *Kdm4b*, *Kdm4d*, *Kdm5c*, *Kdm6a*, and *Kdm6b* compared with the control NT group (Figure 4A), indicating a potential correlation among H4K20me3, H3K9me3, H3K4me3, and H3K27me3. It is reported that bivalent H4K20me3 and H3K4me3 domains are located in intergenic regions and transcriptional start sites of active genes, which exhibits decreased RNA polymerase II pausing and are poised for deactivation of its binding.^8^ This means that repressive mark H4K20me3 can inhibit the expression of those active genes during development and differentiation, while knockdown of its methyltransferase *Suv4‐20h2* promotes the expression of relative development‐dependent genes. Therefore, a combination strategy that overexpresses multiple demethylases KDMs may be better suited to overcome multiple epigenetic barriers (such as H3K9‐, H3K27‐, H4K20me3) and improve cloning efficiency. Despite the three‐fold increase in the blastocyst formation rate and the improved ratio to seven‐fold of cloned mice after *Suv4‐20h2* knockdown, the overall development ratios of NT embryos and cloned mice remained as relatively low as 5.9%. It is possible that donor nucleus may maintain inherently resistant H4K20me3 signatures even after remodelling or reprogramming by oocyte cytoplasm. The detailed regulatory mechanism is not known. Indeed, epigenetic memory is often exhibited by NT embryos. For example, *Xenopus*‐cloned blastula embryos derived from muscle donor nuclei express the muscle gene marker *MyoD* in the neuroectoderm (i.e., nerve/skin cell lineage) and endoderm (i.e., intestine linage) to an excessive extent in about half of all embryos.^37^ Therefore, it is necessary to first erase the H4K20me3 epigenetic memory in donor nuclei by overexpression of *Rad23b* in donor cells or knockdown of *Suv4‐20h2* prior to NT and to subsequently reestablish embryonic H4K20me3 patterns in cloned embryos. In addition, we found knockdown *Suv4‐20h2* can increased mouse cloning efficiency, but the weight of cloned fetus and placenta was significantly higher than that of naturally fertilized mice. We believe that large fetus and placenta syndrome was not corrected through improved H4K20me3 modification probably due to the other inherent epigenetic mechanism.

## 5 CONCLUSION

In summary, our study revealed a dynamic pattern of H4K20me3 epigenetic modification during fertilization, PA, and NT reprogramming. We also demonstrated that aberrant H4K20me3 is an epigenetic barrier to NT reprogramming and that *Suv4‐20h2* knockdown markedly enhanced the blastocyst formation rate of NT mouse embryos and the efficiency of newborn cloned mice by facilitating transcriptional reprogramming. *Suv4‐20h2* knockdown may provide an advantage for efficient transcription at the first cell cycle and for cell fate determination at the blastocyst formation stage (Figure graphical abstract). These findings provide a strategy and a basis for improving the efficiency of cloning and NT reprogramming. Additionally, our results provide insight into the mechanism of H4K20 trimethylation in reprogramming, regulation, and function of cell plasticity during natural reproduction and somatic cell NT in mice.

## AUTHOR CONTRIBUTIONS

Zhihui Liu, Liyou An, and Fuliang Du designed research; Zhihui Liu, Weiguo Wang, Yuhan Xia, and Yuan Gao, Zhisong Wang, Mingyang Li, performed research; Zhihui Liu, Weiguo Wang, and Fuliang Du analysed data; and Zhihui Liu, Liyou An, Giorgio AntonioPresicce, Fuliang Du wrote and revised the manuscript.

## FUNDING INFORMATION

This study was supported in part by the Natural Science Foundation of China (NSFC) Grant Numbers 31872353, 32072732, 31340041, and 31471388 to FD and NSFC Grant Number 31701285 to LA.

## CONFLICT OF INTEREST STATEMENT

The authors declare no conflicts of interest.

## Supporting information


**Figure S1.** Rad23b protein expression in mouse fertilized PA, and NT embryos at different developmental stages.
**Table S1.** RT‐PCR primers used for detecting gene expression.

## Data Availability

The data that support the findings of this study are available from the corresponding author upon reasonable. Data sharing not applicable to this article as no datasets were generated or analyzed during the current study.
