# Supplementary material for: Overcoming the H4K20me3 epigenetic barrier improves somatic cell nuclear transfer reprogramming efficiency in mice
Source: Cell Prolif. 2023 Jun 15;57(1):e13519. doi: 10.1111/cpr.13519 (PMC10771106; doi:10.1111/cpr.13519)
Supplement: Supplementary file 1 — Figure S1. Rad23b protein expression in mouse fertilized PA, and NT embryos at different developmental stages. Table S1. RT‐PCR primers used for detecting gene expression. [file CPR-57-e13519-s001.docx]

**Overcoming the H4K20me3 epigenetic barrier improves somatic cell nuclear transfer reprogramming efficiency in mice**

**Supplementary Data**

**Figure S1. Rad23b protein expression in mouse fertilized， PA and NT embryos at different developmental stages.** (**A**) Fertilized embryo panels: strong Rad23b staining showed in 2-cell (n=22, B1–B2), 4-cell (n=23, C1–C2), 8-cell (n=22, D1–D2), morula (n=20, E1–E2), and blastocyst (n=20, F1–F2), but absent at the 1-cell stage (n=23, A1–A2). PA embryo panels: After activation, oocytes carried none Rad23b staining in two PPN (n=24, A3–A4), Rad23b intensity gradually increased at 2-cell (n=22, B3–B4) 4-cell (n=20, C3–C4), 8-cell (n=19, D3–D4), morula (n=18, E3–E4), and blastocyst (n=20, F3–F4) stage, respectively. NT embryo panels: Rad23b staining was absent in the 1-cell stage (n=24, A5–A6), but gradually increased at 2-cell (n=22, B5–B6), 4-cell (n=18, C5–C6), 8-cell (n=15, D5–D6), morula (n=13, E5–E6), and blastocyst (n=14, F5–F6) stage, respectively. Scale bar = 40 μm. (**B**) Values of a and b among the same embryo stage indicated statistically significant differences (*P* < 0.05). The mean intensity of Rad23b in fertilized embryos was significantly higher than that in both NT and PA embryos at 2-cell, 4-cell, 8-cell, morula, and blastocyst stage, respectively. Arrow-head indicated the nucleus, arrow indicated the polar body nucleus.

Table S1. Sequence of primers for RT-PCR

| **Gene Symbol** | **Forward Primer (5’-3’)** | **Reverse Primer (5’-3’)** | **PCR Condition** |
| --- | --- | --- | --- |
| *Suv4-20h2* | GCTTCCGCACCCATAAGATGA | AGGTCCCGTTGCCTTAGGA | 95℃ 15 s，Annealing/Extension 60℃ 60 s, 40 Cycles |
| *Rad23b* | ACCTTCAAGATCGACATCGACC | ACTTCTGACCTGCTACCGGAA | 95℃ 15 s，Annealing/Extension 60℃ 60 s, 40 Cycles |
| *Dux* | GAGCCCACCACACACATTCT | GGGGAACAGATTCTGGCAGT | 95℃ 15 s，Annealing/Extension 60℃ 60 s, 40 Cycles |
| *Zscan4* | GGTTACCTCTTCCTCCCACTCCAGG | TGTGCCGTTAATGGCCGTGCC | 95℃ 15 s，Annealing/Extension 60℃ 60 s, 40 Cycles |
| *Hmgpi* | GTGGTGAGCATCTTCGGAGTGG | GCGTAGGATTCGTAGGCGTTGT | 95℃ 15 s，Annealing/Extension 60℃ 60 s, 40 Cycles |
| *Klf5* | GCATCGCCCACAGCCCATCACT | CTGGTTGGACGAACAGAACTTGA | 95℃ 15 s，Annealing/Extension 60℃ 60 s, 40 Cycles |
| *Kdm4b* | ACCTATGCGGACAATTCATC | CAGACCAGAGACTGGGATAC | 95℃ 15 s，Annealing/Extension 60℃ 60 s, 40 Cycles |
| *Kdm4d* | AGATTGAAGCCCTCAAAGG | AGCTTGACGTTCATAAGCTC | 95℃ 15 s，Annealing/Extension 60℃ 60 s, 40 Cycles |
| *Kdm5c* | GACCCATCGCCGAGAAGTC | TCGGGGAGTAAACCTGAAGTT | 95℃ 15 s，Annealing/Extension 60℃ 60 s, 40 Cycles |
| *Kdm6a* | CGGGCGGACAAAAGAAGAAC | CATAGACTTGCATCAGATCCTCC | 95℃ 15 s，Annealing/Extension 60℃ 60 s, 40 Cycles |
| *Kdm6a* | AGTGAGGAAGCCGTATGCTG | AGCCCCATAGTTCCGTTTGTG | 95℃ 15 s，Annealing/Extension 60℃ 60 s, 40 Cycles |
| *β-actin* | GCATTGTTACCAACTGGGACG | GACCAGAGGCATACAGGGACAG | 95℃ 15 s，Annealing/Extension 60℃ 60 s, 40 Cycles |
